# Single‐cell transcriptomic analyses of skin vascular endothelial cells in sedentary and voluntary wheel running young mice

**DOI:** 10.14814/phy2.70537

**Published:** 2025-09-05

**Authors:** Pegah Hejazi, Pauline Mury, Éric Thorin, Guillaume Lettre

**Affiliations:** ^1^ Montreal Heart Institute Montreal Quebec Canada; ^2^ Department of Medicine, Faculty of Medicine Université de Montréal Montreal Quebec Canada; ^3^ Department of Surgery, Faculty of Medicine Université de Montréal Montreal Quebec Canada

**Keywords:** endothelial cells, endothelial functions, exercise, physical activity, single‐cell, *Zbtb16*

## Abstract

Physical activity (PA) is a fundamental aspect of preventive medicine, offering profound benefits for cardiovascular health and overall well‐being. Despite its widespread benefits, the molecular mechanisms underlying PA‐induced improvements in microvascular functions remain poorly understood. The skin microvasculature is uniquely affected by exercise‐induced shear stress, especially during thermoregulation. We assigned 20 mice to either a sedentary group or a 1‐month voluntary exercise program involving running wheels. We assessed endothelial function in mesenteric arteries and found no significant difference between groups, consistent with prior reports of minimal vascular effects from short‐term PA in young healthy mice. Post‐intervention, we collected skin biopsies from 12 mice for single‐cell transcriptomic analyses. The differential expression analysis showed a significant difference in the expression of the Zbtb16 gene in vascular endothelial cells (vECs), with higher levels in the physically active group. Additionally, Gene Set Enrichment Analysis (GSEA) with nominally differentially expressed genes in vECs highlighted the suppression of pathways related to oxidative stress, cell proliferation, and metabolism in the exercise group. This suggests an exercise‐triggered transition towards improved metabolic efficiency and enhanced homeostasis in vECs. These results begin to reveal transcriptomic differences in vECs of the skin microvasculature between physically active and sedentary mice.

## 1 INTRODUCTION

Regular physical activity (PA) significantly reduces mortality rates, decreases hospitalization frequency, and enhances the quality of life for all individuals, including those with cardiovascular diseases (Anderson et al., 2016). Longitudinal research has shown that maintaining or adopting an active lifestyle significantly lowers the risk of all‐cause and cardiovascular disease mortality in patients with coronary heart disease, emphasizing the importance of PA trajectories in long‐term health outcomes (Gonzalez‐Jaramillo et al., 2022). Despite the known health benefits, the molecular mechanisms that explain the protective effects of PA are still not clearly understood, especially the differences among interindividual responses.

Recent studies have shown the positive impact of PA on microvascular functions. An 8‐week aerobic training program improved microcirculation reactivity and endothelial function in the skin of patients with ischemic heart disease (Szyguła et al., 2020). An elevated risk of coronary heart disease is associated with impaired endothelium‐dependent vasodilation and reduced capillary recruitment in the skin, suggesting that skin microvascular function serves as an effective model for exploring the relationship between cardiovascular risk factors, microvascular health, and PA (IJzerman et al., 2003).

High‐throughput single‐cell RNA‐sequencing (scRNAseq) methods provide unique insights at cellular resolution, revealing cellular heterogeneity, differential gene expression responses, and cellular functions within highly organized tissues. These studies have already uncovered vascular cell heterogeneity in the human skin, showing that vascular endothelial cells (vECs) are more active in intercellular crosstalk rather than merely serving as passive components of the vascular lining (Jovic et al., 2022; Li et al., 2021). vECs are particularly relevant to monitor PA‐induced changes in transcriptomic profiles of the skin microvasculature. It is known that elevated shear stress is the main signal that triggers endothelial adaptation to exercise, and these changes are inclusive not only to the muscle microvascular system but also to other organs such as the skin (Padilla et al., 2011). During exercise, the blood flow of the working muscles increases and is primarily directed to the skin to enable thermoregulation (Simmons et al., 2011). Different studies also support the notion of exercise‐induced changes in vascular responsiveness of the skin (Crandall et al., 2003). For instance, Wang and collaborators detected improved vascular endothelial responses, specifically enhanced endothelium‐dependent dilation in the skin vasculature, following an 8‐week PA training program in healthy men, with these effects reversing to the pretraining state upon detraining (Wang, 2005).

These findings prompted us to examine the transcriptomic profile of the murine skin microvasculature between physically active and sedentary mice to better understand the cellular mechanisms underlying microvascular function. We focused on the cellular heterogeneity of the skin microvascular network and how exercise influences endothelial function, aiming to uncover pathways through which PA promotes vascular health. This research may provide insights into the mechanisms that modulate vascular health through PA and contribute to the development of targeted therapeutic strategies for improving the microvascular state.

## 2 METHODS

### 2.1 Animals

Twenty wildtype C57Bl/6 mice were included in this project. Among them, 10 were male and 10 were female; all were aged 5 months. All animal experiments were performed in accordance with the Guide for the Care and Use of Experimental Animals of the Canadian Council on Animal Care and the Guide for the Care and Use of Laboratory Animals of the US National Institutes of Health (NIH Publication No. 85–23, revised 1996). Experiments were approved by the Montreal Heart Institute Ethics Committee (ET No. 2019‐42‐01). Mice were kept under standard conditions (24°C; 12‐h:12‐h light/dark cycle) and were fed ad libitum with regular chow (2019S; Harlan Laboratories, Madison, WI, US).

### 2.2 Physical activity intervention

Ten mice (5 male and 5 female) were randomly assigned to the “Physical Activity” (PA) group and were exposed to 1 month of voluntary exercise. To this end, mice were kept individually in cages instrumented with a running wheel (Lafayette Instrument Company, Lafayette, IN). Each running wheel was equipped with a counter to track the running activity of each individual mouse. The remaining 10 mice were also housed individually in standard cages (i.e., without running wheels) and served as the sedentary (SED) control group. Mice were weighed at the beginning and end of the PA month. Mice were sacrificed at 6 months after anesthesia with a 1:1 mixture of xylazine (Bayer Inc., Toronto, ON, Canada) and ketamine hydrochloride (Bioniche, Belleville, ON, Canada) at the same time of the day (morning).

### 2.3 Free fatty acid plasmatic level assay

To assess a physiological difference between the PA and SED groups at the end of the intervention, we assessed free fatty acid plasma levels using the Free Fatty Acid Quantification Colorimetric/Fluorometric Kit (BioVision, Milpitas, CA; Cat# K612‐100). These measurements were conducted post‐intervention only. Each sample was assayed in duplicate, and the mean of the two values was used for analysis.

### 2.4 Vascular reactivity

Two 2‐mm long segments of freshly collected mesenteric arteries were mounted in a wire myograph filled with 10 mL of physiological salt solution, as previously described (Thorin et al., 2000). Mesenteric arteries were selected because they are a widely used model for assessing systemic vascular reactivity in mice due to their accessibility, reproducibility, and suitability for wire myograph experiments. Two arteries per mouse were mounted, and the best‐quality recording was chosen for analyses. We recorded isometric changes in tension: arterial segments were pre‐constricted with phenylephrine (PE; 10 to 30 μM; Sigma, Cat# P‐6126); at the plateau of the constriction, segments were relaxed by cumulative addition of increasing concentrations (from 1 nM to 10 μM) of acetylcholine (ACh; Sigma, Cat# 420–150) to assess endothelium‐dependent relaxation. At the end of the experiment, the segment was maximally constricted with 127 mM KCl‐physiological solution (NaCl replaced by KCl to induce maximal depolarization of the vascular smooth muscle cells) to calculate the percent of constriction induced by phenylephrine. The concentration of ACh inducing 50% of relaxation (ACh‐EC_50_), indicative of the vascular sensitivity to ACh, as well as the maximal relaxation (E_max_), was calculated to characterize endothelial function.

### 2.5 Statistical analyses for physiological data

At first, the normality of distribution was assessed using the D'Agostino & Pearson (D'agostino & Pearson, 1973) test between physically active (PA) and sedentary (SED) groups. Normally distributed data were compared using a parametric unpaired two‐tailed Student's *t*‐test (body weight change, plasma free fatty acid levels, and endothelial maximal relaxation E_max_) otherwise, a nonparametric Mann–Whitney test was used (endothelial sensitivity EC_50_). Data are presented as mean ± standard deviation (SD) for Student's *t*‐test and median [Q1–Q3] for Mann–Whitney test. *p* value <0.05 was considered statistically significant. All analyses were performed using GraphPad Prism (version 9).

No formal a priori sample size analysis was performed for this study. The number of animals used per group was based on commonly accepted standards in preclinical vascular and single‐cell transcriptomic studies, as well as ethical and logistical considerations. We acknowledge that the limited number of animals, particularly for the scRNA‐seq subset, may reduce statistical power to detect subtle physiological or transcriptomic changes.

### 2.6 Skin biopsy and scRNAseq library preparation and sequencing

Upon sacrifice, a piece of skin from the leg was harvested and immediately processed as detailed below. A subset of 12 mice (6 from each group and sex) was used for single‐cell transcriptomic profiling. Two pieces of 1 cm^2^ of skin were harvested per mouse and were dissociated using the Whole Skin Dissociation Kit (Miltenyi Biotec, Germany, Cat# 130–101–540) with some variations regarding the manufacturer's instructions. Skin tissue was incubated at 37°C in a water bath for 1 h 30 in the enzyme mix. Mechanical dissociation was then processed using the ThermoMixer (Eppendorf) for 1 h at 37°C and 400 rpm. Following completion of the dissociation, the homogenate was filtered through a 70‐μm cell strainer into a 50‐mL centrifuge tube. The cell suspension was then centrifuged at 600*g* for 10 min at 4°C. Once complete, the supernatant was removed and the pellet was incubated in 500 μL of Red Blood Cell Lysis Buffer for 1 min 30 (Roche, Cat# 11814389001). Cells were again centrifuged at 600*g* for 10 min at 4°C and then resuspended in 500 μL of PBS + 0.04% BSA. Cell count and viability were estimated using the Countess II FL cell counter (Thermo Fisher).

Endothelial cells were then enriched using the CD31 MicroBeads mouse Kit (Miltenyi Biotec, Germany, Cat# 130–097‐418), following the manufacturer's instructions. Briefly, cells were incubated for 30 min with 10 μL of beads. After a wash step, cells were loaded onto an MS column on the OctoMACS Separator. The enriched fraction of CD31+ cells was recounted using the Countess II FL cell counter (Thermo Fisher) and were immediately loaded onto a 10X Chip and processed on the 10X Chromium Controller. Samples were sequenced at Genome Quebec's Centre d'Expertise et de Services on an Illumina NovaSeq sequencer with a PE100 protocol.

### 2.7 Data processing and quality‐control

FASTQ files were aligned to Cellranger's mm10‐2020‐A reference using the count function of cellranger‐7.0.1 for each sample (https://www.10xgenomics.com/support/software/cell‐ranger/latest/release‐notes/cr‐reference‐release‐notes). To remove the ambient RNA contamination, we used the SoupX tool (Young & Behjati, 2020). For downstream analyses, we used Seurat v4 (Hao et al., 2021). We removed low‐quality cells using the following thresholds: >200 and <5000 detected genes, mitochondrial reads <20, ≥500, and <40,000 sequence reads, RNA complexity >0.8. To normalize and regress out mitochondrial read percentage, we used SCTransform (Hafemeister & Satija, 2019). We integrated the data using Harmony on 30 principal components (Korsunsky et al., 2019). To annotate cell types, we obtained the skin canonical markers from two different databases: *Tabula muris* (Schaum et al., 2018) and immunesinglecell skin atlas (Li et al., 2022). We plotted the expression levels of key marker genes obtained from both databases onto the UMAP representation of our dataset to assign celltype to each cluster. We identified doublets using scDblFinder (Germain et al., 2022). We removed cells for which the scDblFinder score was >0.5. We sub‐clustered each main cell type and removed subclusters showing doublet enrichment.

### 2.8 Differential gene expression analysis

To identify differentially expressed genes (DEGs) across different condition groups for each cell‐type, we conducted a pseudobulk analysis. This approach involves aggregating gene expression counts at the sample level within each cell‐type to create bulk‐like profiles for each condition. We utilized Seurat's AggregateExpression() function to perform this aggregation, effectively summarizing the expression data for each cell‐type across all samples. We used a likelihood ratio test (LRT) implemented in DESeq2 to perform differential expression analysis (Love et al., 2014). The model included group (PA vs. SED) as the primary variable and sex as a covariate. This allowed us to identify genes that are differentially expressed in the group of mice subjected to PA compared to those that were not. Genes with a false discovery rate (FDR) <5% were significantly associated with PA.

### 2.9 Gene set enrichment analysis (GSEA)

To gain insights into the biological pathways and processes associated with the DEGs in vECs, we performed GSEA (Subramanian et al., 2005). To employ GSEA, we used the complete output from our differential gene expression analysis performed with DESeq2. This approach included both statistically significant and nonsignificant genes ranked based on a combined metric of their *p* values and log_2_ fold change, allowing us to account for both the statistical significance and the direction of expression changes. We employed the fgseaSimple() function from the fgsea package (Korotkevich et al., 2021) to conduct the GSEA, leveraging the hallmark gene sets from the Molecular Signatures Database (MSigDB) collection specific to mouse genes. We used the Spearman's correlation test to identify genes that are nominally differentially expressed in vECS and correlated with the expression of *Zbtb16* (adjusted *p* value <0.05).

## 3 RESULTS

### 3.1 Body weight and free fatty acid levels

**FIGURE 1:**
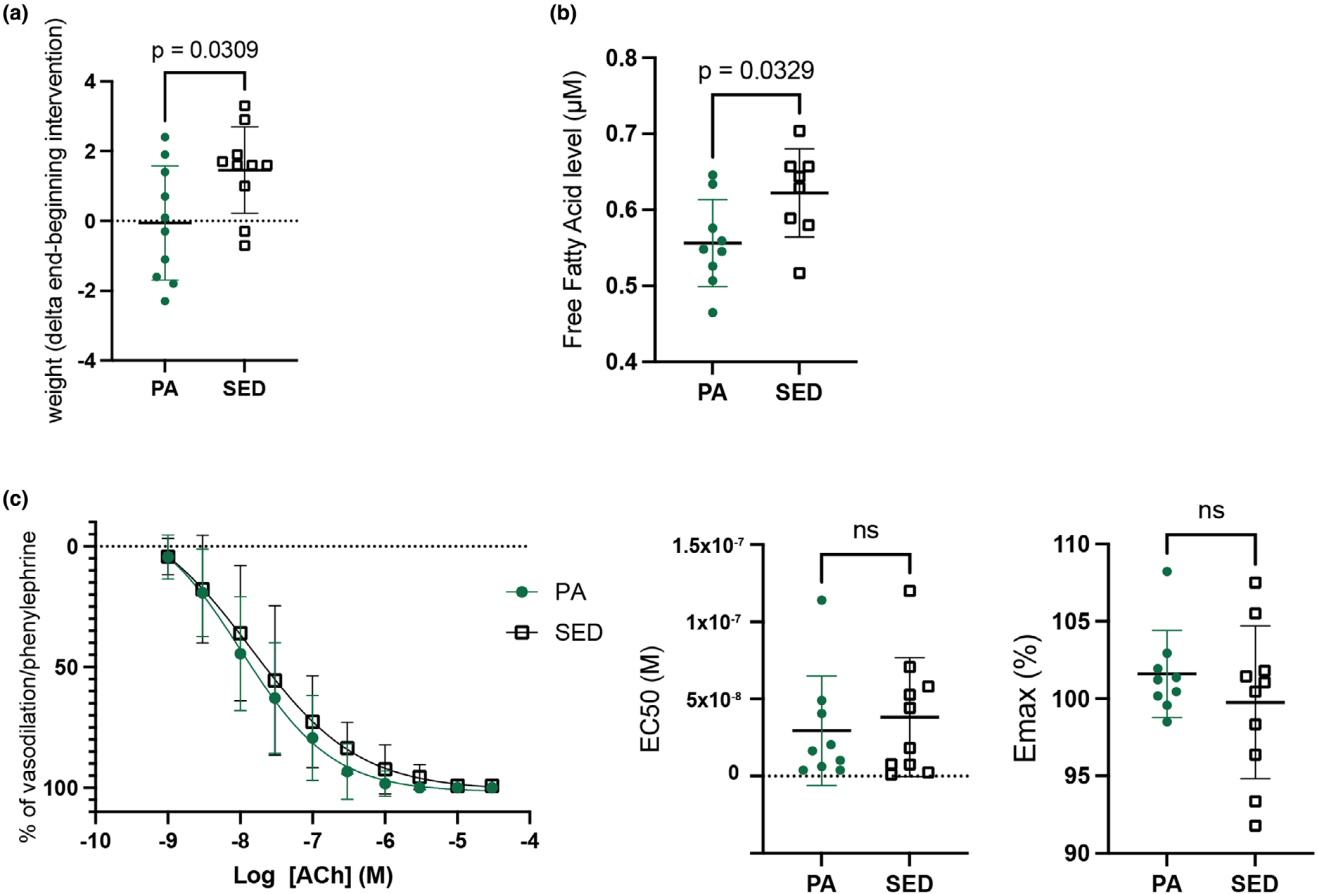
Group differences in body weight, free fatty acid levels, and vascular reactivity. (a) Evolution of body weight from mice in the PA group using a two‐tailed unpaired *t*‐test that is significantly lower compared to mice in the sedentary group (SED) (PA = −0.06 ± 1.64 g vs. SED = 1.46 ± 1.24 g; two‐tailed *t*‐test *p* value = 0.031). (b) Post‐intervention plasma levels of free fatty acid (FFA) using a two‐tailed unpaired *t*‐test are significantly lower in PA mice compared to SED mice (PA = 0.56 ± 0.057 μM vs. SED = 0.62 ± 0.058 μM; *p* value = 0.033). (c) No significant differences were observed in endothelial function as measured by acetylcholine (ACh)‐induced relaxation. Group differences were assessed respectively using a Mann–Whitney test for EC_50_ and an unpaired *t*‐test for E_max_ values. EC_50_ (sensitivity to ACh): PA = 16.4 nM [5.13–44.8 nM] versus SED = 31.2 nM [6.24–61.2 nM], *p* value = 0.66. E_max_ (maximal relaxation): PA: 101.6 ± 2.81% versus SED: 99.8 ± 4.94%; *p* value = 0.34. ns, nonsignificant.

We recorded the voluntary running mileage of all mice with an automated wheel‐attached counter (Table S1). We calculated the total distance run (30 days). The average run distance was 164.4 ± 24.25 km, denoting a large variability between individuals, due to the chosen mode of exercise (voluntary). To validate the efficacy of the intervention, we assessed two different parameters: (1) evolution of body weight before and after intervention and (2) plasma levels of free fatty acid (FFA), post‐intervention (Figure 1). PA mice showed no significant change in body weight compared to baseline (Δweight = −0.06 ± 1.64 g; one‐sample *t*‐test *p* value = 0.91), whereas SED mice gained significant weight (Δweight = +1.46 ± 1.24 g; *p* value = 0.0047). Between groups, body weight change was significantly different (*p* value = 0.031), indicating that PA prevented the weight gain observed in sedentary controls (Figure 1a). Similarly, plasma levels of FFA were significantly lower in PA mice than in SED mice (PA = 0.56 ± 0.057 μM vs. SED = 0.62 ± 0.058 μM; *p* value = 0.033) (Figure 1b). These two results validate our model of exercise.

### 3.2 Vascular reactivity

We measured two indexes of vascular reactivity to assess endothelial function: ACh‐EC_50_ for endothelial sensitivity to acetylcholine (ACh) and E_max_ for the maximal relaxation (Figure 1c). We performed the vascular reactivity analysis in each of the 20 mice, and then compared the effect of the exercise treatment. Voluntary exercise did not improve neither endothelial sensitivity (EC_50_) to ACh (PA: 16.4 nM [5.13–44.8 nM] vs. SED: 31.2 nM [6.24–61.2 nM], *p* value = 0.66) nor maximal relaxation (E_max_) (PA: 101.6 ± 2.81% vs. SED: 99.8 ± 4.94%; *p* value = 0.34). This finding suggests that 1 month of voluntary exercise is not sufficiently potent to induce significant changes in mesenteric arterial endothelial function that can be measured in 20 healthy 6‐month‐old mice.

### 3.3 Identification and characterization of cell populations in murine skin

**FIGURE 2:**
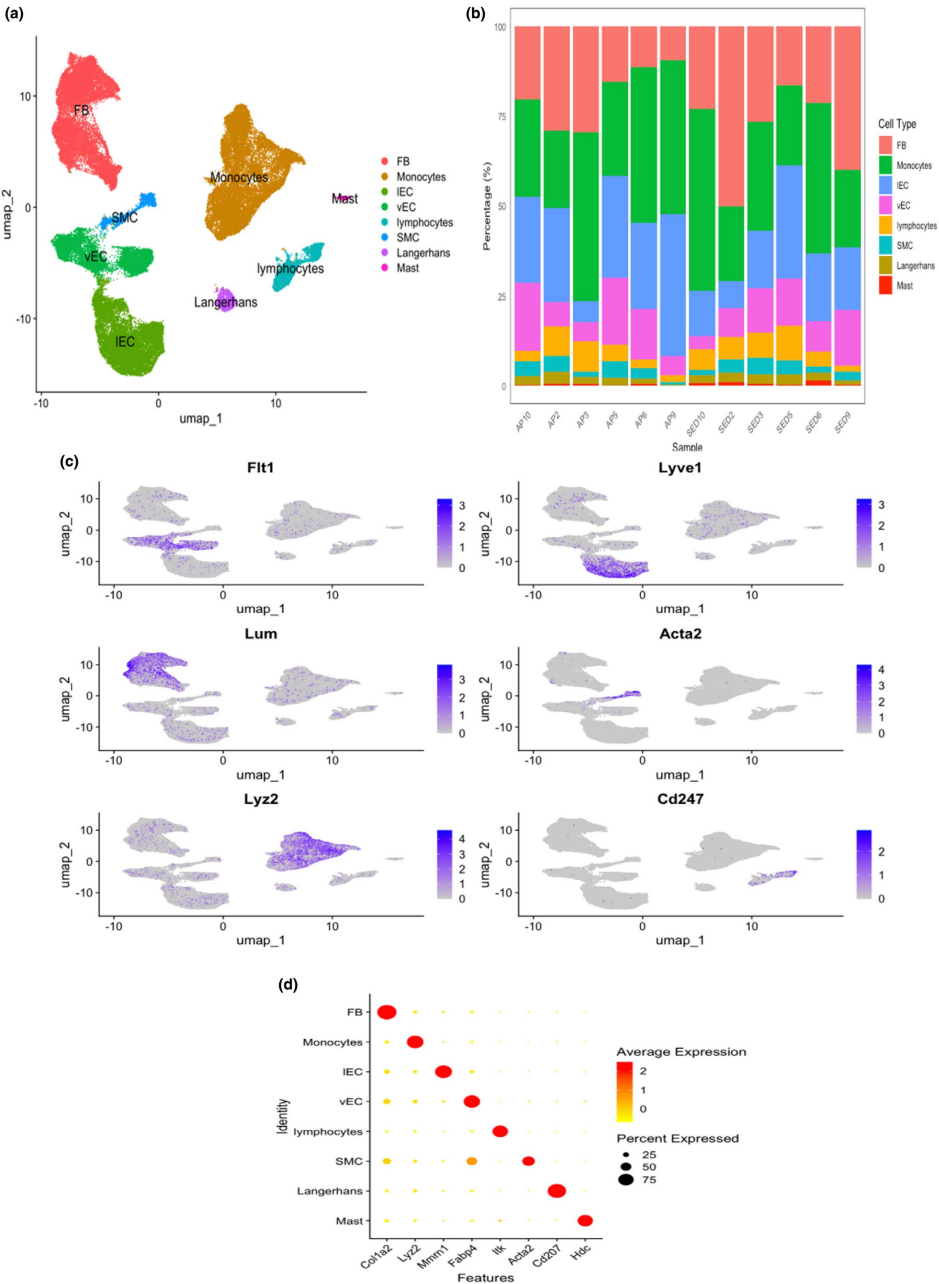
Single‐cell RNA‐sequencing analysis of the mouse skin microvasculature. (a) UMAP visualization of the dataset post‐removal of keratinocytes and basal cells. The unbiased clustering of 66,112 cells displays a variety of vascular and immune cell‐types. Key cell‐types identified in the dataset include vascular endothelial cells (vECs), smooth muscle cells (SMCs), fibroblasts (FBs), lymphatic endothelial cells (lECs), monocytes, mast cells, Langerhans, and lymphocytes. (b) Stacked bar plot illustrating the proportion of different cell‐types across samples. Each bar represents a sample, labeled by sample IDs, with colors indicating different cell types: FB (salmon pink), Langerhans (olive), IEC (light blue), monocytes (green), mast cells (red), SMC (turquoise), lymphocyte (orange), and vEC (pink). The y‐axis shows the percentage of cells, highlighting the distribution and relative abundance of each cell‐type within the samples. (c) The figure presents feature plots for key marker genes specific to each cell‐type used to confirm cluster annotations. The expression of these genes was visualized across the UMAP. Each plot shows the spatial distribution and expression intensity of the marker genes, enabling the validation of cell‐type identities within the clusters. Some of the key marker genes include: *Flt1*: Marker for vECs, *Lyve1*: Marker for lECs, *Acta2*: Marker for SMCs, *Lum*: Marker for FBs, *Cd247*: Marker for lymphocytes, *Lyz2*: Marker for monocytes. (d) Dot plot illustrating the primary marker gene for each cluster by average and percentage of expression.

While we could not detect a physiological change in endothelial function after 1 month of exercise, we wondered whether such treatment could modify gene expression programs. To address this question, we performed scRNAseq on a subset of the same mice. Unsupervised Seurat‐based clustering of 141,226 cells (48% of which are keratinocytes) from the skin of 12 mice revealed nine distinct cell types. After quality‐control steps and the removal of keratinocytes and basal cells to focus on cell types of the microvascular environment, we obtained a single‐cell dataset with 66,112 cells (Figure 2a). This dataset included vECs (5.4%), smooth muscle cells (SMCs, 3.9%), fibroblasts (FBs, 31.8%), lymphatic endothelial cells (lECs, 21.0%), monocytes (31.2%), mast cells (0.6%), Langerhans cells (2.0%), and lymphocytes (4.2%) (Figure 2b).

To confirm cluster annotation, we assessed the expression of marker genes using the uniform manifold approximation and projection (UMAP) (Figure 2c). After sub‐clustering and doublet removal (Section 2), we identified 3625 vECs, our main cell type of interest. These vECs constitute 5.4% of the total cells in our quality‐controlled dataset. This proportion of vECs aligns with expectations for dermal vascular density, considering the removal of major epidermal populations (keratinocytes and basal cells) and using the endothelial cell enrichment method (Section 2).

### 3.4 Transcriptomic analyses of murine skin vECs

**FIGURE 3:**
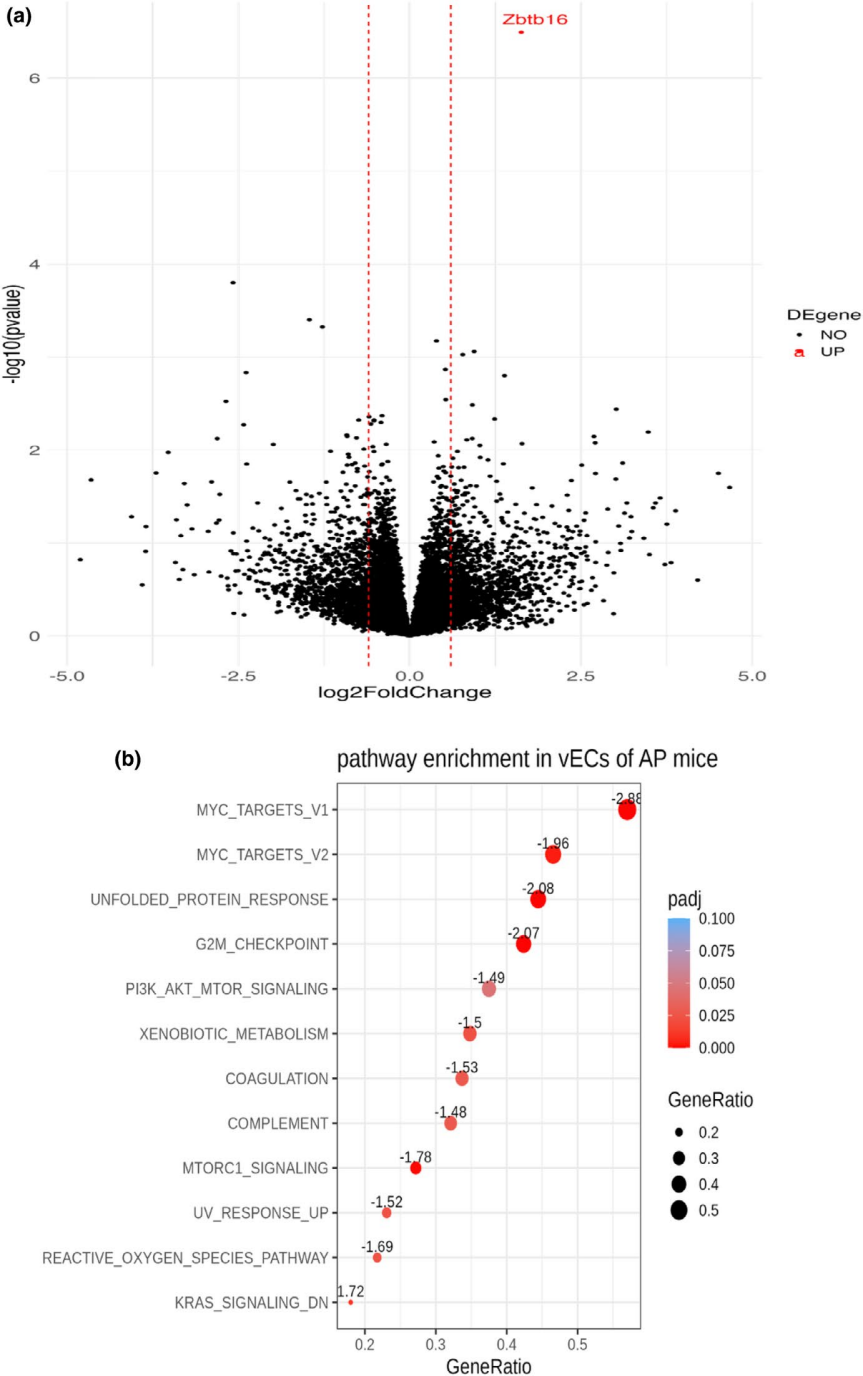
Differentially expressed gene and gene set enrichment analyses. (a) Volcano plot depicting differentially expressed genes. The analysis identified *Zbtb16* (zinc finger and BTB domain containing 16) as significantly overexpressed in vECs of the PA mouse group compared to the SED group after multiple testing correction. (b) Gene Set Enrichment Analysis (GSEA) of vECs from murine skin comparing the PA and SED groups. The analysis shows significant enrichments in pathways related to cell cycle regulation and stress response.

By performing differentially expressed gene analysis in vECs, we found a single gene that was significantly different between the PA and SED groups after multiple testing correction (Table S2). *Zbtb16* (zinc finger and TBT domain containing 16), also known as promyelocytic leukemia zinc finger (PLZF), was significantly overexpressed in vECs of the PA mouse group (Figure 3a). In the other cell types that we profiled by scRNAseq, we found no other genes that were significantly differentially expressed (Table S3). While not the focus of our study given the limited sample size, we also performed sex‐stratified differential gene expression analysis in vECs and found four genes (*Cyp1a1*, *Cfd*, *Car4*, and *Gm11290*) downregulated in female mice from the PA group (Figure S1). The role of these genes in response to PA or cellular stress in vECs is unknown, although Cyp1a1 expression is induced by shear stress (Eskin et al., 2004) and Cfd has been implicated in several cardiovascular and metabolic diseases (Kong et al., 2024).

While we could only detect one gene that was significantly dysregulated in murine vECs upon exercise after correcting for multiple testing, we reasoned that several genes that reached nominal significance could nominate common biological pathways. GSEA highlighted enrichment of several key pathways related to cell cycle regulation, such as the “activation of KRAS‐signaling‐DN” pathway that captures genes that are downregulated when KRAS signaling is activated (Table S4). GSEA results also revealed a consistent suppression of multiple pathways typically associated with cell proliferation (MYC Targets V1 and V2), stress response (Reactive Oxygen Species Pathway, Unfolded Protein Response, and UV Response Up), and metabolism (mTORC1 Signaling) in vECs of PA mice (Figure 3b). The suppression of these pathways suggests a shift towards lower cellular stress in vECs of PA mice. Many genes implicated by the GSEA are correlated with the expression of the transcription factor *Zbtb16* (47 and 8 positively and negatively correlated, respectively, adjusted *p* value <0.05) (Table S5).

## 4 DISCUSSION

In humans, regular PA increases nitric oxide production and bioavailability and reduces the production of pro‐inflammatory cytokines and reactive oxygen species (Di Francescomarino et al., 2009). These observations point towards an effect of PA on endothelial functions, but until recently, it was challenging to distinguish the PA‐triggered molecular changes that occur in vECs from those that occur in other cell types of the blood vessels (e.g., smooth muscle cells and immune cells). In this study, we took advantage of scRNAseq to profile the transcriptome of vECs from the murine skin microvasculature in response to exercise.

Our analyses identified a single gene, *Zbtb16*, that is upregulated in skin vECs after exercise. *Zbtb16* encodes a poorly characterized transcription factor that has not been directly linked to PA response yet. Earlier work suggested that *Zbtb16* can suppress endothelial cell proliferation and angiogenesis (Joko et al., 2017; Rho et al., 2010), and that it is also involved in the endothelial response to various stimuli such as arsenic (Moore et al., 2024), far‐infrared therapy (Hsu et al., 2012), or pro‐inflammatory TNFα treatment (Lalonde et al., 2019). Our analyses showed that *Zbtb16* expression in vECs is correlated with the expression of many genes implicated in biological pathways that respond to exercise (see below). Taken together, these results suggest that *Zbtb16* plays a critical role in vEC adaptation to cellular stress. Further experiments are now needed to dissect whether and how the *Zbtb16* transcriptional network contributes to improving endothelial functions in response to PA.

Acute exercise is generally associated with increased stress response in many tissues (Contrepois et al., 2020). In contrast, our GSEA suggests that a 1‐month PA period leads to a suppression of stress‐related pathways in vECs (Figure 3b). The downregulation of stress response pathways, such as the Reactive Oxygen Species and Unfolded Protein Response pathways, indicates that PA may enhance vEC resistance to cellular stress. The dysregulation of genes implicated in the key mTOR pathway also suggests that the potential increased vEC resilience to stress after PA may be explained by more efficient cell metabolism (Figure 3b). Consistently, *Zbtb16* has been implicated in the metabolic syndrome through an impact on mitochondrial functions, fatty acid oxidation, and glycolysis (Liška et al., 2017; Šeda et al., 2017).

We also conducted exploratory sex‐stratified differential expression analysis and found four genes downregulated in skin vECs of female PA mice. While the functional implications of these differences remain unclear, they raise the possibility of sex‐specific endothelial responses to PA that warrant further investigation.

Body weight was maintained in PA mice, whereas SED mice gained weight over the course of the intervention. Although we did not measure food intake directly, this difference in weight trajectory could contribute to some of the observed transcriptomic adaptations. Weight gain and increased adiposity are known to impair endothelial function in both resistance and conduit arteries by promoting oxidative stress and inflammation (Pierce et al., 2008). While our study was specifically designed to assess the effects of PA rather than diet‐induced weight changes, it is possible that the suppression of stress and metabolic pathways observed in vECs reflects a combination of exercise‐induced effects and the protective impact of preventing weight gain. Future studies should incorporate metabolic markers and food intake measurements to disentangle exercise‐specific from weight‐associated adaptations.

Our study has two main limitations. First, we noted that a voluntary 1‐month exercise exposure in young and healthy mice was not sufficient to improve vascular endothelial functions (Figure 1a,b). No weight loss was observed (Figure 1c). For plasma free fatty acid levels, we did not measure changes over time; instead, only group differences between PA and SED mice were assessed (Figure 1d). Although we did not observe improvements in mesenteric artery endothelial function, this may be due to the short duration of the intervention and the already healthy vascular baseline in young animals. Similar observations have been made in other vascular beds, where short‐term exercise training failed to alter vasoreactivity in young rodents (Donato et al., 2005). It is likely that a longer exercise treatment in mice could highlight other molecular mechanisms associated with the vascular benefits of PA. Second, despite our attempt to enrich for endothelial cells (Section 2), vECs only represented 5.4% of all the cells that we profiled by scRNAseq, which is expected considering that the endothelium is a monolayer in blood vessels. This resulted in lower statistical power to identify genes for which PA has a modest impact on their expression levels. Because the skin is notoriously difficult to dissociate—an essential step in scRNAseq experiments—the development of more robust protocols to prepare skin samples will improve the yield of such experiments for rarer cell‐types (Waise et al., 2019).

In this study, we used young and otherwise healthy mice to characterize baseline transcriptomic changes in skin vascular endothelial cells associated with physical activity. While this approach reduced potential confounding factors, it also limited our ability to detect changes in endothelial function, which are more prominent in aging or disease models. Future studies using aged mice or models of vascular dysfunction will be essential to determine whether the transcriptional patterns observed here, such as *Zbtb16* expression, are preserved, amplified, or altered in those contexts.

In conclusion, we profiled a transcriptomic dataset of the skin of voluntary exercising and sedentary mice, focusing on vECs. Our aim was to identify differentially expressed genes regulated by PA. We found that the transcription factor *Zbtb16* is highly upregulated in the skin vECs of PA mice and may participate in the adaptation of cellular stress. Because the skin is easily accessible, our study highlights the feasibility of extending this experimental protocol to better understand the vascular response of human subjects to PA.

## AUTHOR CONTRIBUTIONS

All authors conceived and designed the experiments. P.M. and P.H. collected the data and performed analyses. E.T. and G.L. secured funding and supervised the work. P.H., P.M., and G.L. wrote the manuscript with contributions from all authors.

## CONFLICT OF INTEREST STATEMENT

The authors declare that they have no competing interests.

## ETHICS STATEMENT

All animal experiments were conducted in accordance with the Guide for the Care and Use of Experimental Animals of the Canadian Council on Animal Care and the Guide for the Care and Use of Laboratory Animals of the US National Institutes of Health (NIH Publication No. 85‐23, revised 1996). All procedures were approved by the Montreal Heart Institute Ethics Committee (protocol number ET 2019‐42‐01).

## CODE AVAILABILITY

The code used to analyze the data and generate the figures is available upon request.

## Supporting information


Figure S1.

Tables S1–S5.

## Data Availability

The scRNAseq data discussed in this publication have been deposited in NCBI's Gene Expression Omnibus and are accessible through GEO Series accession number GSE295877 (https://www.ncbi.nlm.nih.gov/geo/query/acc.cgi?acc=GSE295877).
