# Supplementary material for: Single‐cell transcriptomic analyses of skin vascular endothelial cells in sedentary and voluntary wheel running young mice
Source: Physiol Rep. 2025 Sep 5;13(17):e70537. doi: 10.14814/phy2.70537 (PMC12413401; doi:10.14814/phy2.70537)

**Supplementary Figure 1.** Volcano plots of sex-stratified differential gene expression analysis of vascular endothelial cells of physically active (wheel running) versus sedentary young mice.


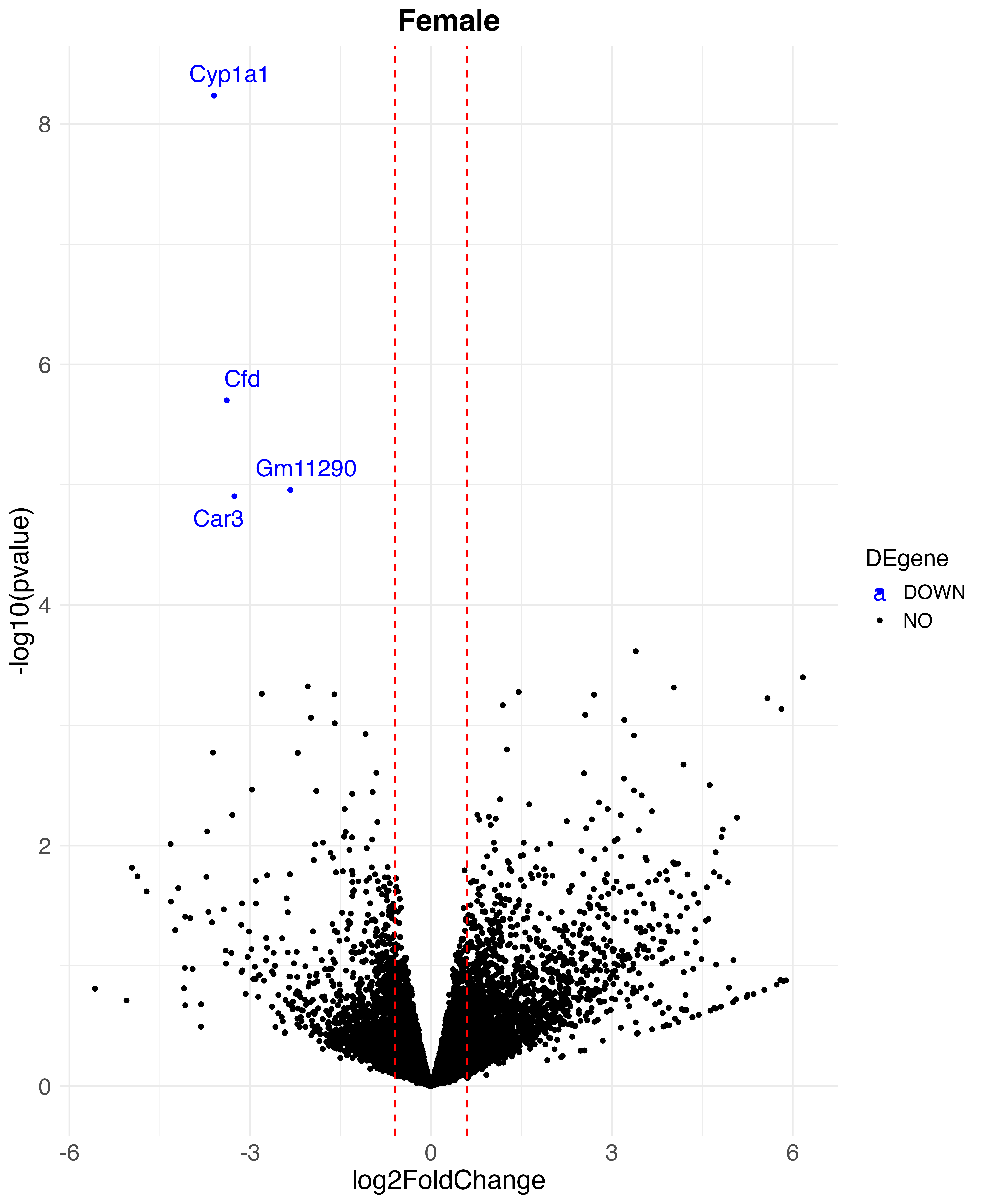


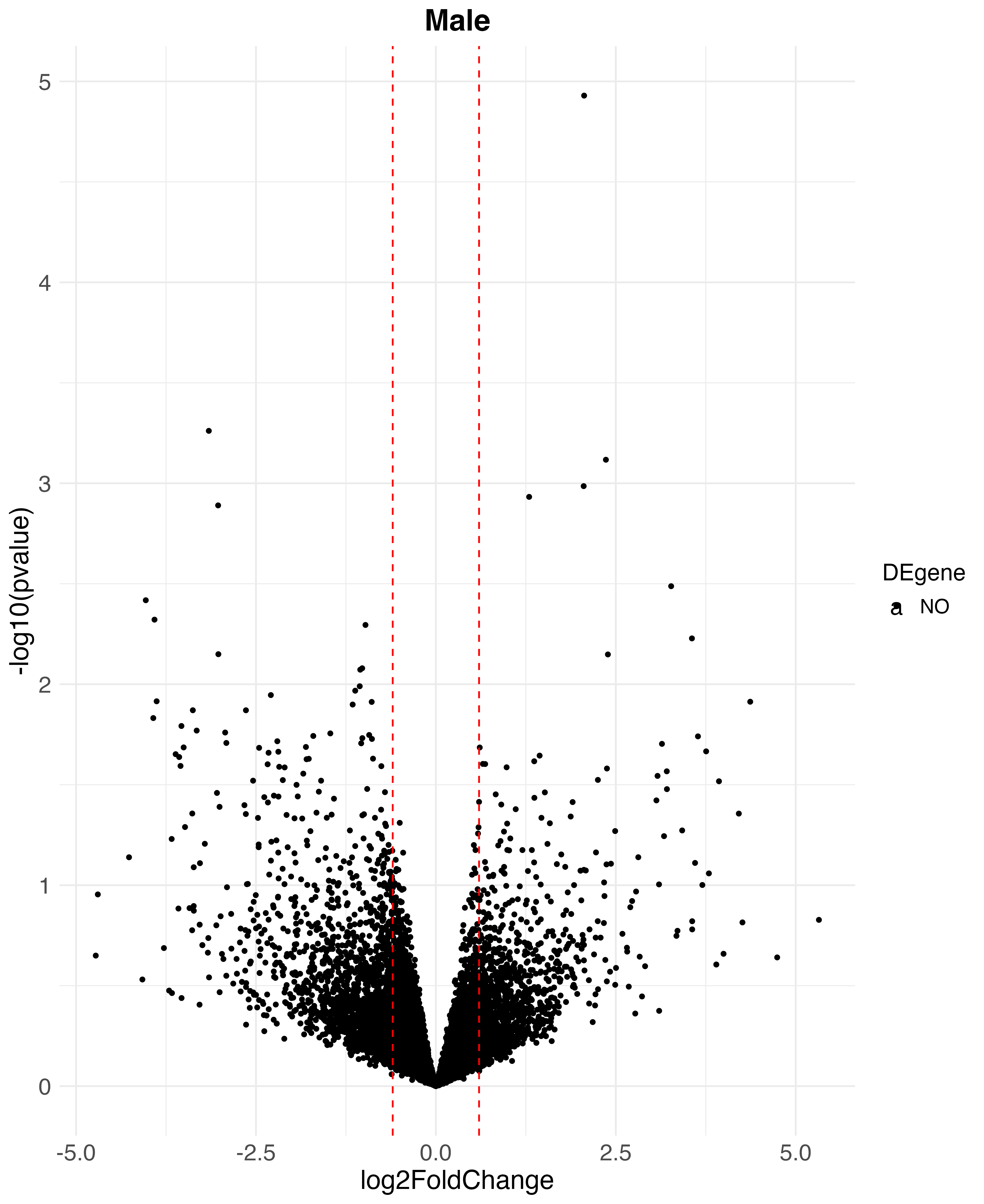

Supplement: Supplementary file 1 — Figure S1. [file PHY2-13-e70537-s002.docx]
